# pmTM-align: scalable pairwise and multiple structure alignment with Apache Spark and OpenMP

**DOI:** 10.1186/s12859-020-03757-2

**Published:** 2020-09-29

**Authors:** Weiya Chen, Chun Yao, Yingzhong Guo, Yan Wang, Zhidong Xue

**Affiliations:** 1 School of Software Engineering, Huazhong University of Science and Technology, Wuhan, 430074 China; 2 School of Life Science, Huazhong University of Science and Technology, Wuhan, China

**Keywords:** Pairwise structure alignment, Multiple structure alignment, Apache Spark, OpenMP

## Abstract

**Background:**

Structure comparison can provide useful information to identify functional and evolutionary relationship between proteins. With the dramatic increase of protein structure data in the Protein Data Bank, computation time quickly becomes the bottleneck for large scale structure comparisons. To more efficiently deal with informative multiple structure alignment tasks, we propose pmTM-align, a parallel protein structure alignment approach based on mTM-align/TM-align. pmTM-align contains two stages to handle pairwise structure alignments with Spark and the phylogenetic tree-based multiple structure alignment task on a single computer with OpenMP.

**Results:**

Experiments with the SABmark dataset showed that parallelization along with data structure optimization provided considerable speedup for mTM-align. The Spark-based structure alignments achieved near ideal scalability with large datasets, and the OpenMP-based construction of the phylogenetic tree accelerated the incremental alignment of multiple structures and metrics computation by a factor of about 2–5.

**Conclusions:**

pmTM-align enables scalable pairwise and multiple structure alignment computing and offers more timely responses for medium to large-sized input data than existing alignment tools such as mTM-align.

## Background

The three-dimensional structure of protein plays an important role in providing inference of its molecular function, and is more conserved than sequence during evolution. Structure comparison can be used to identify functional and evolutionary relationship between proteins, which are very useful for functional annotation, structure-based drug design, protein–protein docking, and many other applications [1].

To compare two protein structures, we first need to find the best structural alignment between two proteins to initiate residue-level comparison. Many pairwise structure alignment (PSA) methods are developed in this aim, like DALI [2], CE [3], TM-align [4], etc. PSA can be further generalized to three or more structures, which is noted as multiple structure alignment (MSTA). As a natural extension of PSA, MSTA can be built with progressive merging of PSA results [5], or based on iterative fragment alignment and assembly [6].

The task of finding alignment without a priori knowledge of equivalent residues is in general an NP-hard problem with no exact solution, which usually involves computation intensive procedures such as dynamic programming. With the dramatic increase of protein structural data in the Protein Data Bank, computation time quickly becomes the bottleneck for large scale structure comparisons. For example, it would take several days to compare a newly solved protein structure iteratively against all existing ones stored in a large database, and even longer to build MSTA with a smaller group of proteins. As a consequence, there is an increasing demand for structure alignment tools that can not only provide accurate results, but also complete large-scale requests with reasonable response time.

To tackle this problem, many newly developed structure alignment tools employ parallel computing to improve the computation efficiency. These parallel computing tools differ drastically both in hardware and software architecture, ranging from multi-core CPU, GPU to cloud platforms.

GPU is suited for fine-grained parallel tasks executed on parallel threads, for example, ppsAlign [7] utilizes a hybrid inter- and intra-task parallel model to divide each PSA task into several independent seed alignments on different threads, while each block executes one or more PSA tasks. GPU-CASSERT [8] adopts a similar two-phase alignment strategy to ppsAlign and implements both the coarse and detailed alignment algorithms on the GPU with an extended set of structural features. Another GPU-based alignment tool named GPU-DALIX [9] achieves a speedup up to 20× over the sequential version of DALIX [10] by using a two-level parallel algorithm for the dynamic programming. These GPU-based tools are able to achieve good performance gain in alignment tasks, though limited by the capacity of a single GPU. Besides GPU, there are also other tools designed for dedicate hardware, e.g. the RckAlign [11] that runs on Single-Chip Cloud Computer from Intel. All these methods require a quasi-full redesign of original alignment tools in order to fit specific architecture.

Cloud computing arises as another efficient computational framework for data intensive problems, it offers outstanding performance and scalability for processing large amount of data that are difficult to be handled by a single computer. Cloud computing has already been used to solve various biological problems [12, 13], for example, the classic Hadoop framework and its MapReduce paradigm have been combined with sequencing tools for different types of sequences such as DNA, RNA and proteins [14]. Hadoop has also been applied to solve structural bioinformatic problems, and there are already some researches on Hadoop based protein structure alignment [15]. For example, Hung and Lin [16] implemented an alignment—refinement scheme for protein structure alignment using MapReduce with DALI and VAST [17], Mrozek et al. developed Cloud4PSi [18] and later HDInsight4Psi [19] based on Hadoop/HBase clusters deployed in Microsoft Azure public cloud. Another MapReduce-based computational solution, called H4P [20], was recently developed using Map-only processing pattern for efficient mining of similarities in 3D protein structures.

More recently, Apache Spark has emerged as a promising and more flexible framework for the implementation of highly scalable parallel applications based on in-memory cluster computing. To maintain the scalability and high fault tolerance, Spark uses a distributed memory abstraction called Resilient Distributed Dataset (RDD), which offers a performance boost against Hadoop's MapReduce. For example, SparkSW [21] is a spark-based sequence alignment tool which directly implements the classic Smith-Waterman algorithm with Scala as part of the Spark driver. SparkBWA [22] is designed for DNA sequence alignment which uses existing BWA tools as internal libraries and calls them with the JNI mechanism. Other parallel computing tools such as SparkBLAST [23] and Spark-IDPP [24] employ Spark pipe to call standalone binaries at each worker node to achieve their goals. There are also many other Spark-based sequencing tools such as PASTASpark [25], MetaSpark [26], SpaRC [27], etc. (a comprehensive review can be found in [28]). However, to the best of our knowledge, no protein structure comparison tools have so far been implemented with Spark.

In this paper, we propose pmTM-align, a parallel protein structure alignment approach based on mTM-align/TM-align. We optimized two major steps in mTM-align with parallel computing tools: Spark-based PSA computation and OpenMP-based incremental alignment. pmTM-align is built on a hybrid architecture to take advantage of data-level parallelism by cloud computing, and fine-grained parallelism of multi-core CPU. The goal is to provide near real-time responses for pairwise and multiple structure alignment requests with large-scale input.

## Implementation

### Design overview

mTM-align is built with progressive merging of PSA results provided by TM-align. It has three main steps: (1) generation of PSAs and a distance matrix by TM-align, (2) construction of a structure-based phylogenetic tree by UPGMA [29], and (3) progressive build of a MSTA by NWDP [30]. We focus on step 1 and 3 for efficiency optimization since they are the most time-consuming steps.

In this aim, we took a closer look on the computations involved in these steps. The PSA generation step mainly concerns grouping protein structures into pairs, then each pair is processed by TM-align in a sequential order. Since there is no dependency between protein pairs, these pairwise computations can be deployed on a distributed system to improve data efficiency.

Once the PSAs and phylogenetic tree is ready, the final multiple alignment is built by progressive merging of intermediate alignment results from leaf nodes to the root. Aligning an already constructed alignment with another alignment/structure is performed using the NWDP algorithm. This task is essentially recursive as alignments at a given non-leaf node will be feasible only when its children nodes have been parsed. This process can be parallelized if the tree is not entirely skewed so nodes at the same depth can be processed simultaneously.

**Fig. 1:**
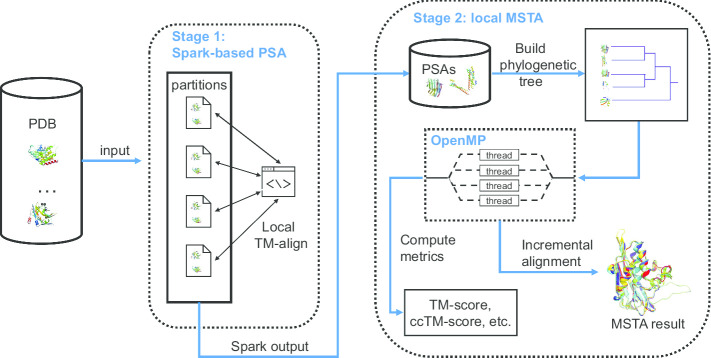
The two-stage design of pmTM-align. In stage 1, pairwise structure alignments (PSAs) are achieved on a Spark cluster. Next, another script was used to collect results from HDFS and then send to a local program for multiple structure alignment

Based on the above analyses, we divide the algorithm into two computational stages depending on their type of dominant task: the iterative PSA stage and the incremental alignment stage. The whole application thus utilizes a hybrid architecture to solve the task in each stage (see Fig. 1). For the PSA stage, we decide to deploy the task on a distributed system and choose Spark as the computing framework for its efficiency and scalability against other frameworks when dealing with large dataset. However, the incremental alignment stage is not suitable for distributed systems because the interdependence of subtasks would induce lots of transmission of alignment results among worker nodes. For the incremental alignment stage, PSA results are downloaded automatically from HDFS to a multi-cores computer, then a program is started to proceed multiple structure alignments with OpenMP.

### Spark-based pairwise structure alignment

Apache Spark outperforms Hadoop's MapReduce scheme for iterative data processing with its in-memory calculations by introducing an elastic distributed data structure abstraction (i.e. Spark RDD). Although Spark can be run in standalone mode and read data from the local file system, we choose to use Hadoop's task scheduling and data management systems, i.e. YARN and HDFS, to have a more integrated distributed computing environment.

**Fig. 2:**
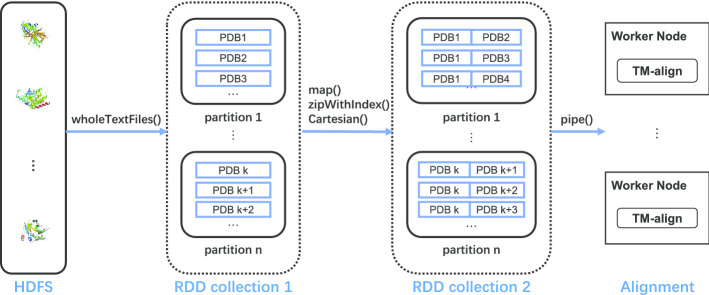
The data workflow of the Spark-based PSA program. RDDs are formed by protein structures loaded from HDFS, and then send to Spark worker nodes for alignment computing

A driver application receives user commands and manage the whole application execution, including data distribution, task execution, and the gathering of results from all worker nodes. The workflow of the PSA stage is as follows: protein structures are first uploaded to HDFS, then are read by the Spark program and transformed to RDDs. HDFS allows data sharing without per site data replication and ensures high throughput when Spark fetches data from it. The RDD collection is then divided into partitions and distributed to each worker node for PSA computation. The final result is collected and saved on HDFS, then downloaded as a single file as input to form the distance matrix. The detailed workflow is shown in Fig. 2. It should be noted that protein structures are grouped into pairs before sending for alignment tasks.

To integrate TM-align into the Spark platform, we chose a pipe-based architecture to call the TM-align program written in C++ as an external library. The procedure is shown in Algorithm 1: first, the *wholeTextFiles* function on line 2 reads all protein structures from files on HDFS, and then the *cartesian* function on line 7 creates protein pairs in a Spark RDD, which are transferred to the local TM-align with a *pipe* operator on line 13, and all comparison results of TM-align are finally collected from standard output to form a new RDD. The input and output of the original TM-align is redesigned to fit the IO requirements of Spark.
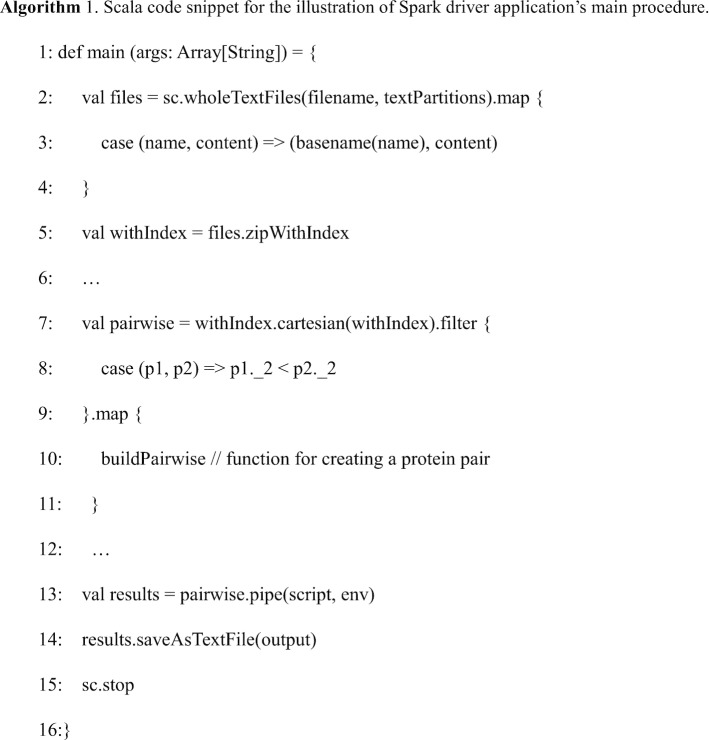


This pipe operation has three advantages over other binding options. First, it requires minimum adaption of the original TM-align program, instead of writing wrapper code like JNI calls. Second, the Spark driver and the command line alignment tool is loosely coupled, so it is highly flexible and open to other alignment algorithms. Finally and more importantly, TM-align is computation intensive with heuristic search and dynamic programming, it is more efficient to stay with C/C++ implementation than having a Scala replicate.

This Spark-based PSA implementation with TM-align can be easily deployed on a local cluster or public cloud with Spark's default parameter settings. However, several parameters are to be fine-tuned if we want to further optimize the computation efficiency.

### Multi-thread computing with OpenMP

#### Data structure optimization

The phylogenetic tree used in the original mTM-align is based on an array-type data structure without direct link from a node to its direct children, each node can be accessed using its index and a string storing the name of the protein structure or a concatenation of names for non-leaf nodes. Although this representation is more compact in memory and allows quick sequential access to all nodes, it is neither efficient for node searching, nor an ideal data structure for tree traversal.

**Fig. 3:**
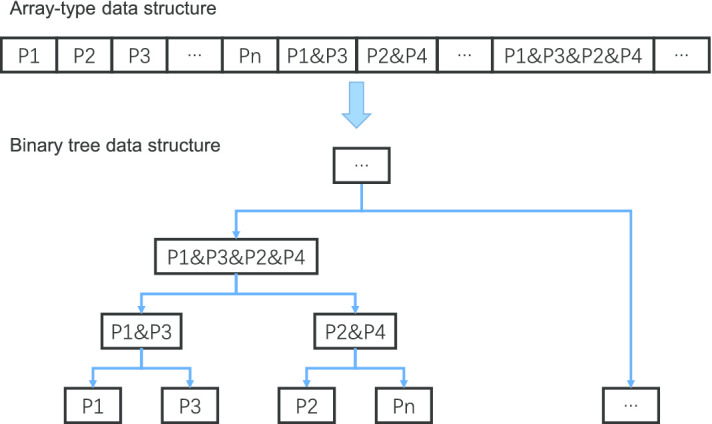
Data structure optimization. The nodes of phylogenetic tree are stored with a binary tree structure instead of an array

So here a binary tree is used instead to store the generated phylogenetic tree, and the index of each node is kept to indicate whether it's a leaf node or not (non-leaf nodes have an index larger than the number of input protein structures). With the binary tree data structure, the incremental alignment order of structures can be generated with standard post-order tree traversal (see Fig. 3).

#### Parallel tree traversal

The post-order traversal of the phylogenetic tree can be parallelized by OpenMP Tasks or Tasking which was introduced in OpenMP 3.0 [31]. OpenMP requires to insert some pragmas into the code to mark the section that should be executed by multiple threads, then tasks will get spread (approximately) evenly among all threads. The running process is managed by a task scheduler in a dynamic manner and transparent to the programmer: if one thread has a backlog of tasks to do, its tasks can be reassigned to other idle threads; tasks can be executed immediately or be deferred, the execution order is only ensured by task synchronization.

In the case of post-order tree traversal, the pseudo code is shown in Algorithm 2. Starting from the root of the phylogenetic tree, each time the function *Traversal* is called, two new threads will be created respectively (by using “omp task” in front of the parallel region) for the processing of children nodes (except for the leaf nodes who have no children), the alignment computation on the current node starts only when its children finish their job on new threads (assured by the keyword *taskwait*). The pragma *single* prevents each thread to do a full traversal and *taskwait* assures the tree traversal order. A global *cutoff* parameter can be defined to limit excessive task subdivision, for example, *cutoff* = 3 means all nodes that have a distance to the root node larger than 3 will not create new threads for their children nodes.
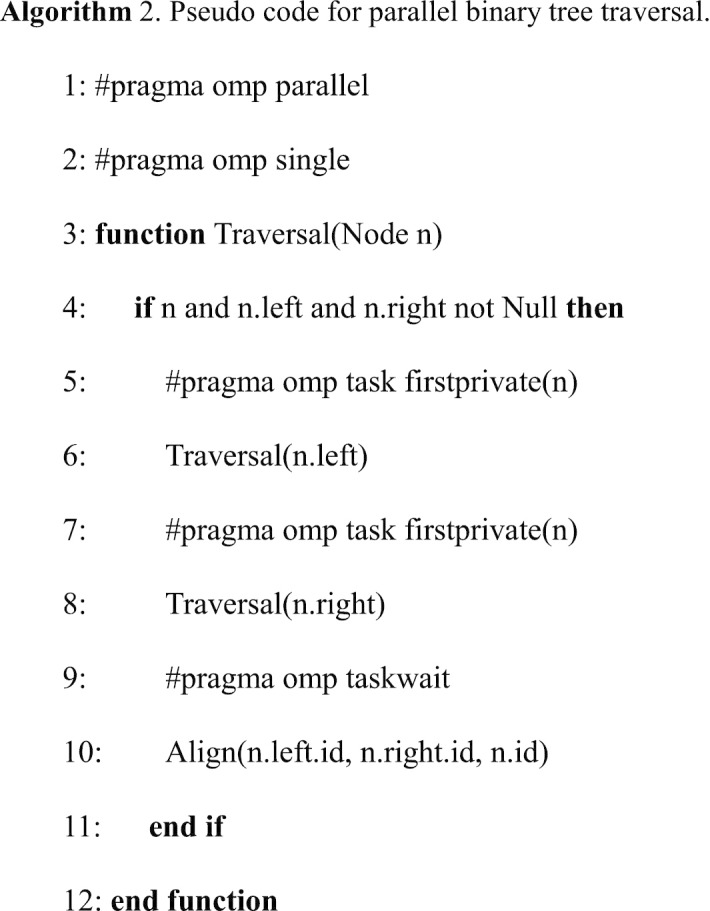


#### Loop schedules

The incremental alignment and metrics computation steps have more than 20 for loops to compute the final alignment and various scores like the average RMSD and TM-score, so they can be easily accelerated by using OpenMP's *for* pragma, the pseudo code is shown in Algorithm 3. The *schedule* clause can be used to control the assignment of loop iterations to the threads. The scheduling method can either be *static* or *dynamic*, we choose the latter for load balancing since protein structures usually have different lengths.
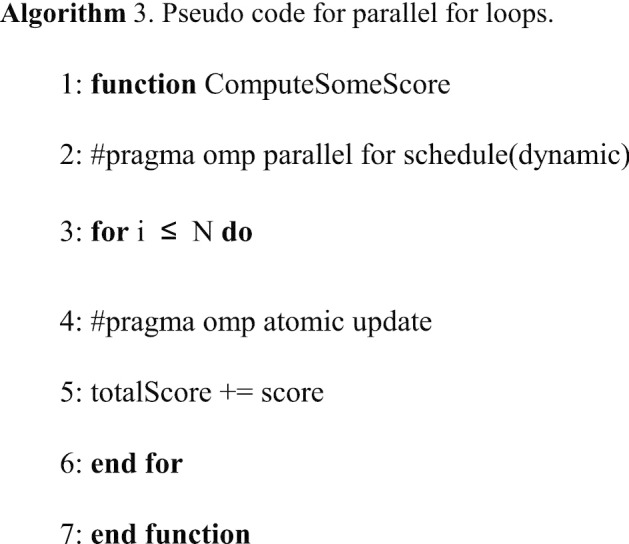


## Results

In order to evaluate the benefits brought by pmTM-align, we conducted a series of experiments to quantify the performance gain in term of computation efficiency. Since pmTM-align contains two stages that are run with different tools, i.e. Spark and OpenMP, we designed the experiment accordingly in a two-stage manner. First, we assessed the speedup ratio for the Spark-based PSA at each input data scale under different Spark settings. Second, with the best Spark configuration identified in previous step, pmTM-align was compared against mTM-align to observe the overall speedup.

### Dataset

We chose the SABmark dataset (version 1.65) [32] for benchmark tests, which is a resource for alignments of sequences with very low to medium sequence similarity. SABmark contains two subsets: superfamily and twilight zone, each having groups of structures with pairwise sequence identity < 50% and < 25% respectively. SABmark is frequently used as a standard benchmark for MSTA algorithms.

To test the scalability of pmTM-align for PSA tasks, we merged all groups of structures in SABmark and created randomly formed groups with different sizes. Start from 25, the size of the test set doubles until reaching 1600 structures. To test the full MSTA process, we also created more fined subsets from 20 to 100 with an interval of 20.

### Hardware and software setup

Experiments were carried out on China Telecom's e-Cloud. Each server instance had an Intel(R) Xeon(R) Gold 6151 CPU@3.00 GHz with 32 cores, 128 GB RAM. Given three server instances, we created a master node and three worker nodes containing 32 Spark executors. Each executor had two cores and 4 GB memory distributed over the worker nodes.

We tested pmTM-align on Centos 7.6 using Spark 2.3.3 along with Hadoop 2.8.5 for its HDFS and YARN components. OpenMP was directly included in the GNU compiler and the version of G++ was 4.8.5.

### Test on spark-based PSA

Given a database of protein structures, there are usually two types of pairwise comparison scenarios: (1) 1-to-all comparison: a query structure is compared against all the rest to find similar structures to the query protein; (2) all-to-all comparison: exhaustive comparisons are performed on the whole database to find potential connections among sequence divergent proteins. The computation tasks involved in these two scenarios are indeed of the same nature, but the second one has a time complexity of O(n^2^) while the first one is O(n). In this study, we mainly focus on all-to-all comparison as a main component of MSTA.

Our preliminary tests show that computing time can not only be influenced by the number of cores in the cluster, but also the number of partitions of Spark RDDs. So in this section we first present performance tests with different number of RDD partitions, then with a chosen partition configuration, we progressively add executors to conduct the scalability test.

### Number of partitions

Spark manages data using partitions that help parallelize distributed data processing with minimal network traffic for sending data between executors. A partition represents a logical chunk of a Spark RDD. We used *wholeTextFiles* instead of *textFile* to read data from HDFS to preserve the relation between file name and content inside by loading the data into a *PairRDD*. The default configuration of *wholeTextFiles* creates only two partitions, which could be suboptimal as it leaves many cores unused. Here we tested the impact of number of input partitions with 1-to-all PSA tasks because the *cartesian* transformation in all-to-all tasks would change the number of partitions.

We launched a series of 1-to-all PSA tasks with subsets of 100, 400 and 1600 structures on all 32 executors. As we assumed, the default setting created only two partitions with up to 1600 structures. As shown in Table 1, the default setting is largely outperformed by user-specified number of partitions no matter the size of input data. Since there are totally 64 cores in the cluster, adding more partitions than the number of cores does not further improve the computation efficiency, on the contrary, will generate more overheads for task scheduling. As long as there are fewer partitions than the number of cores, Spark is not able to automatically repartition the RDD to make use of free cores.

**Table 1:** The impact of input partitions

Number of input partitions	Total execution time			Average execution time per pair		
	Subset100	Subset400	Subset1600	Subset100	Subset400	Subset1600
Default (2)	25.54	42.29	97.28	0.25	0.11	0.06
64	22.61	22.12	26.41	0.22	0.06	0.02
128	21.57	23.95	25.69	0.22	0.06	0.02
256	22.93	23.69	25.6	0.23	0.06	0.02

Total execution time (in s) and average execution time per pair (in s) are computed for 1-to-all PSA tests

As mentioned above, the number of partition is not fixed in an all-to-all PSA task, the *cartesian* product transformation that generates structure pairs can potentially create lots of partitions, for example, an input dataset of 100 structures can lead to more than 2000 partitions during execution, which is over subdivided as each partition will contain less than five structure pairs. To deal with over subdivision, we can apply *coalesce* operation to force the fusion of partitions to a given number. So here we test the impact of partition fusion by all-to-all PSA tasks with subsets of 100, 400 and 1600 structures on all 32 executors. The initial partition number is set to 64 with or without partition fusion.

The results in Table 2 show that partition fusion with the *coalesce* operation can to some extent improve the computation efficiency against the default Spark behavior. However, the number of partitions after fusion should not be too small with respect to the scale of input computational tasks, otherwise the computation time will grow dramatically (e.g. 128 partitions with subset 400 and 1600).

**Table 2:** The impact of partition fusion

Number of partitions after fusion	Total execution time			Average execution time per pair		
	Subset100	Subset400	Subset1600	Subset100	Subset400	Subset1600
64	93.69	936.09	14,128.78	0.02	0.01	0.01
128	35.60	234.91	13,136.66	0.01	0.003	0.01
320	35.02	145.92	1702.24	0.01	0.002	0.001
640	36.71	135.97	1666.90	0.01	0.002	0.001
960	39.89	140.88	1696.40	0.01	0.002	0.001
1280	37.89	140.74	1652.48	0.01	0.002	0.001
Default^a^	42.46	144.38	1728.53	0.01	0.002	0.001

Total execution time (in s) and average execution time per pair (in s) are computed for all-to-all PSA tests

^a^The default number of partitions are 2025, 3364 and 3969 for subset 100, 400 and 1600 respectively

### Scalability test

Scalability test is performed by varying both the number of executors and the scale of input data to see how the Spark-based PSA implementation performs with different size of workload and how the performance evolves with increasing computing resources.

We conducted a series of tests with fixed number of input partitions (64) and increasing number of executors (from 2 to 64) to plot the speedup ratio and parallel efficiency for all-to-all PSA tasks.

**Fig. 4:**
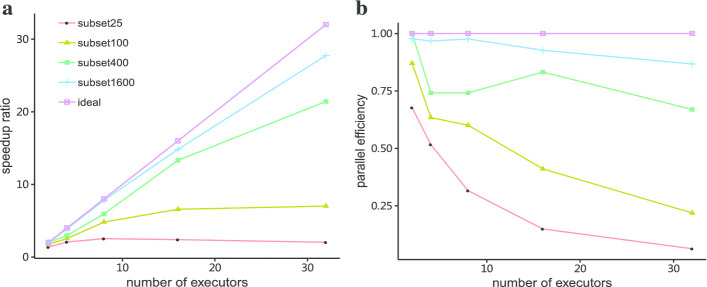
Results of scalability tests by running all-to-all PSA tasks on 2 to 32 executors. **a** The speedup ratio; **b** parallel efficiency

As shown in Fig. 4a, the speedup ratio of PSA tasks scales almost linearly with the number of executors when the input data reaches about 400, and comes close to ideal speedup ratio with an input of 1600 structures. However, when there are very few structures to be aligned (around 20), it is not useful, even less efficient to deploy the task to more Spark executors. Regarding the parallel efficiency, or the speedup per processor, it drops with increasing number of executors as more overheads like communication cost are involved, although we observe some non-linearity at 16 executors for subset 400 (see Fig. 4b).

From the above results, we can see that except for very small input data, the Spark-based PSA program is able to have good computing efficiency and scales well with different size of datasets. However, it's not trivial to choose an “optimal” number of executors for all tasks. Since parallel efficiency is not a prioritized concern, a good choice would be using all available computing cores to gain as much speedup as possible.

### Test on end-to-end MSTA

With the best Spark configuration identified in previous section, we proceed the comparison between pmTM-align and the original mTM-align to see the actual benefits of parallel computing for end-to-end MSTA tasks. Here end-to-end means taking a group of structures as input, and giving the final alignment and associated scoring metrics as output. pmTM-align was benchmarked with and without OpenMP so we can see the contribution of Spark and OpenMP separately. pmTM-align without OpenMP (i.e. single-threaded) is referred as *pmTM-align-single* hereafter for simplicity.

The three methods were compared using randomly sampled structures from the SABmark dataset. As the computation load for all-to-all PSA grows quadratically with the number of structures and mTM-align may provide invalid results for large number of randomly selected proteins if their structures are too divergent to find common core regions, here we mainly used small subsets to illustrate the performance gain of our modified versions.

**Fig. 5:**
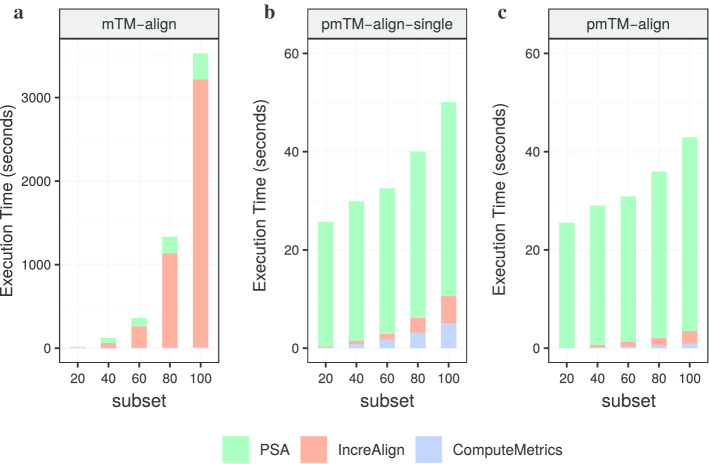
Total execution time for MSTA tasks. The performance benchmark on total execution time running MSTA tasks with **a** mTM-align, **b** single-thread pmTM-align, **c** pmTM-align

The results for subsets containing 20–100 structures are presented in Table 3. We can see that the total execution time was largely reduced with pmTM-align-single and pmTM-align for subsets with more than 40 structures. pmTM-align is the fastest method in most cases, though it got very similar performance with pmTM-align-single. The difference between these methods grows as the dataset becomes larger. In the best case of all tests, a 72-fold gain in performance can be achieved with subset 100. However, the modified versions were not as good as mTM-align when dealing with very small input data, e.g. subset 20. To better understand the exact source of performance gain or loss with different input data, we further analyzed the execution time of each method's major steps and the result is plotted in Fig. 5.

**Table 3:** The benchmark on execution time (in s) for MSTA tasks

Dataset	mTM-align	pmTM-align-single	pmTM-align
Subset20	17.66	25.81	25.73
Subset40	123.54	30.36	29.82
Subset60	361.87	33.41	32.22
Subset80	1335.21	41.59	39.04
Subset100	3531.87	55.79	49.02

Regarding mTM-align, the most time-consuming steps are all-to-all PSA and incremental alignment, the latter can take up to 90% of total running time (see Fig. 5a). These two steps are both accelerated, as a result of parallel adaptation and data structure optimization.

**Fig. 6:**
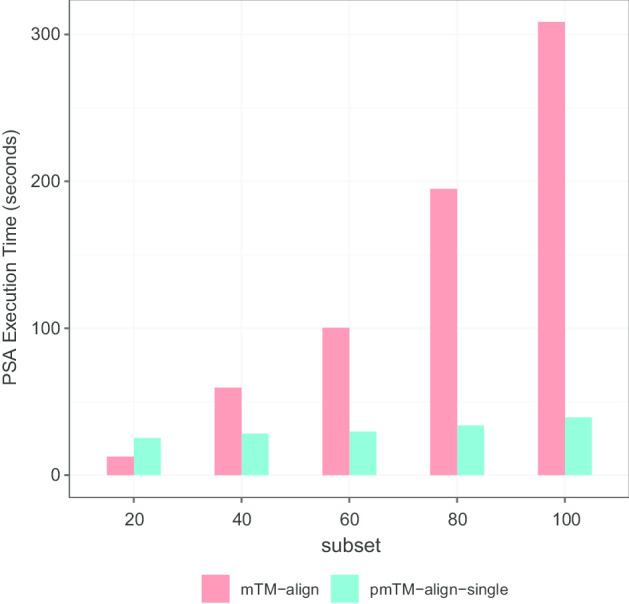
The benefit of Spark-based PSA. Comparison of PSA execution time between mTM-align and pmTM-align-single

To further understand the benefits of each modification that we apply to the original mTM-align, we first compared mTM-align with pmTM-align-single to see the influence of Spark and altered data structure. We can see in Fig. 5b, c that, Spark has indeed helped to largely improve the computation efficiency for all-to-all PSAs. While mTM-align needs more than 300 s to process a small dataset with 100 structures which generates 4950 pairs, pmTM-align-single only needs around 40 s (about sevenfold boost). Figure 6 also shows it's not worth launching Spark when there are very few data to be processed. Besides the acceleration provided by Spark, optimization for phylogenetic tree with binary tree and ID-based indexing methods also had a great impact on the computing efficiency.

**Fig. 7:**
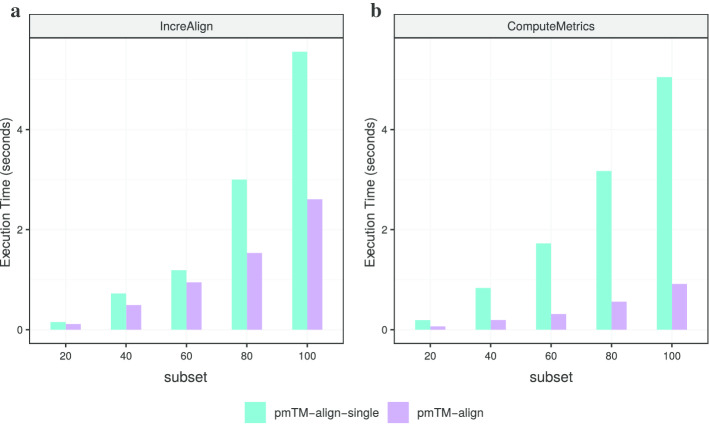
The benefit of multi-thread computing with OpenMP. Comparison between pmTM-align-single and pmTM-align on execution time for **a** incremental alignment, **b** metrics computing

Then to assess the benefits of OpenMP, we compared pmTM-align-single and pmTM-align by their execution time on incremental alignment (Fig. 7a) and metrics computation (Fig. 7b). Results show that both steps can be further accelerated despite the huge improvements that we already got from data optimization in pmTM-align-single. Here all 32 cores on a single server were allocated by OpenMP and the best speedup ratio was about 2 and 5.5 for incremental alignment and metrics computation respectively.

## Discussion

From the results presented in previous section, we confirmed the benefits of using Spark and OpenMP for MSTA tasks. The final program named pmTM-align now allows datasets of medium or large sizes to be processed in a reasonable time. pmTM-align is divided into two computational stages and each stage is accomplished by the corresponding computing environment forming a hybrid architecture.

The Spark-based PSA part yields significant improvements for TM-align based alignment tasks compared to the original serial version. The results of scalability tests (see Fig. 4) show that the Spark-based all-to-all PSA program can have both good speedup ratio and parallel efficiency when the size of input data exceeds a certain limit (e.g. over 400 structures). We observed some irregularities with subset 400: the parallel efficiency had a peak at 16 executors. The exact cause is unclear, a possible source could be the distribution of executors on our specific hardware setup.

As Spark is essentially designed to handle large datasets, when dealing with smaller datasets, we can still have acceptable speedup ratio, but the parallel efficiency drops quickly. From the scalability analyses, we recommend a configuration of no more than 16 executors (each has 2 cores) when dealing with less than 100 input structures.

A specialty about this Spark-based PSA tool that has been previously discussed is the input partition issue. Since each structure file in the SABmark dataset is about 20 KB, Spark will only create two partitions if there are less than 12,000 structures to be loaded from HDFS. This default behavior leads to unbalanced workloads among executors, and thus impede efficient task execution. As shown in Table 1, the default setting can get similar results with user-specified conditions for about 100 structures, but this inefficiency quickly grows for more input data. So normally the number of input partitions can be left to its default value, but should be set to the number of cores available in the cluster for large input dataset.

It should be noted that, many Hadoop-based PSA tools exist, but were not compared in this study for the lack of support for TM-align. It is thus impossible to draw conclusions on the comparison between Hadoop and Spark for PSA tasks based on the reported results in this study. Further comparisons are needed to run PSA on the same hardware, with the same alignment algorithm and datasets.

Regarding OpenMP, the loop parallelism with *for* pragma worked well and is the main source of performance gain shown in Fig. 7. Although the incremental alignment and metrics computation are no longer bottleneck steps for MSTA tasks after data structure optimization, the involvement of OpenMP is still useful when dealing with large datasets. However, the Task-based parallel tree traversal didn't provide any visible acceleration, it's sometimes even longer than the serial version. We believe there are two main reasons for this result: first, the computation complexity increases from leaf to root nodes as more structures need to be aligned together when “climbing” the tree, but parallelism appears mostly at leaf nodes. Second, the actual speedup ratio depends also on the shape and size of the binary tree, which is determined by the scale and structural similarity among the input proteins. In the best case, the tree is fully balanced, a theoretical speedup ratio of (n − 1)/(2*h) can be reached, where n is the number of nodes and h = log_2_ (n + 1)—1 is the tree's height. In the worst case, the tree is skewed in a way that alignment can only be built one after another. We observed the shape of phylogenetic trees built from our randomly sampled subsets and some groups in SABmark superfamilies set who share a common evolutionary origin, but no apparent rules can be find for the shapes of phylogenetic trees from these two different sources. So it's recommended to keep the loop parallelism and remove the Task-based parallel tree traversal.

Finally, the optimal combination of design choices led us to Spark-based PSA plus parallel loops with OpenMP for doing MSTA tasks.

## Conclusion

In this paper, we propose pmTM-align, which largely improves the computing efficiency of mTM-align by applying data structure optimization and integration of parallel computing tools including Apache Spark and OpenMP. pmTM-align enables scalable pairwise and multiple structure alignment computing and offers more timely responses than mTM-align, it can not only process small to medium-sized data, but also has the potential to handle very large datasets if not limited by physical memory.

Currently pmTM-align employs a hybrid two-stage architecture as Spark can only handle the all-to-all PSA part, while the rest is computed locally with OpenMP support. In the future, efforts will be made to find ways to build phylogenetic tree and achieve incremental alignment in a distributed environment, or to combine Spark and GPU to further improve the computing efficiency for PSA tasks.

## Availability and requirements

- *Project name*: pmTM-align.
- *Operating system(s)*: Centos 7.6 or other Linux distributions.
- *Programming language*: Scala, Shell and C++.
- *Other requirements*:Spark 2.3.3 or higher, Hadoop 2.8.5 or higher, G++ 4.8.5 or higher, OpenMP 3.0.
- *License:* BSD 3-clause Clear License.
- *Any restrictions to use by non-academics*: No extra restrictions except BSD 3-clause Clear License.

## Data Availability

The datasets generated and/or analysed during the current study are available in the SABmark repository, https://bioinformatics.vub.ac.be/databases/databases.html.

## Abbreviations

PSA: Pairwise structure alignment
MSTA: Multiple structure alignment
RDD: Resilient distribution dataset
HDFS: Hadoop distributed file system
